# Utility of High-frequency Jet Ventilation in Atrial Fibrillation Ablation

**DOI:** 10.19102/icrm.2021.120708

**Published:** 2021-07-15

**Authors:** Savalan Babapoor-Farrokhran, Jafar Alzubi, Zachary Port, Ola Khraisha, Sumeet K. Mainigi

**Affiliations:** ^1^Department of Medicine, Division of Cardiology, Einstein Medical Center, Philadelphia, PA, USA; ^2^Sidney Kimmel Medical College, Thomas Jefferson University, Philadelphia, PA, USA

**Keywords:** Atrial fibrillation, catheter ablation, general anesthesia, high-frequency jet ventilation, radiofrequency ablation

## Abstract

Atrial fibrillation (AF) is the most common clinically significant arrhythmia that causes major morbidity and mortality. Catheter ablation focusing on pulmonary vein isolation is increasingly used for the treatment of symptomatic AF. Advances in ablation technologies and improved imaging and mapping have enhanced treatment efficiency but only modestly improved the efficacy. Another—but less commonly used—technology that can have a favorable impact involves enhancing the catheter–tissue contact by manipulating respiration to promote improved catheter stability and optimal contact. High-frequency jet ventilation (HFJV) is a mode of ventilation that can reduce respiratory movements to almost apneic conditions. In this review article, we aimed to highlight different studies, review the current literature regarding the utility of HFJV in AF ablation, and discuss the safety and efficacy of this approach relative to that of conventional ventilation.

## Introduction

Atrial fibrillation (AF) is the most common clinically significant arrhythmia and causes major morbidity and mortality.^1,2^ Catheter ablation with pulmonary vein isolation (PVI) is a well-established rhythm-control strategy used in the treatment of symptomatic AF.^3^ Data from large registries have indicated a reduction in all-cause mortality, risk of stroke, and heart failure hospitalizations in patients who underwent AF ablation.^4^ Supporting these conclusions, data from both the Catheter Ablation Versus Standard Conventional Therapy in Patients with Left Ventricular Dysfunction and AF (CASTLE-AF) and Catheter Ablation Versus Antiarrhythmic Drug Therapy for AF (CABANA) trials showed that there is both a mortality benefit and a reduction in AF hospitalization in patients who undergo catheter ablation.^5,6^ As compared with antiarrhythmic drug therapy, catheter ablation has both a better efficacy profile and a lower complication rate in patients with paroxysmal and persistent AF.^7,8^ The procedure is typically performed using either radiofrequency (RF) energy or, less commonly, by cryoablation.^9^ Advancements in three-dimensional mapping and intracardiac echocardiography have led to a reduction in the use of fluoroscopy and radiation exposure.^10^ Despite technological advancements in ablation catheters and the development of new procedural techniques, the long-term success rates of the procedure remain suboptimal.^11^ Manipulating respiration to improve catheter stability and tissue contact is an underutilized technique to improve procedural efficacy.^12^

High-frequency jet ventilation (HFJV) is a mode of ventilation that reduces diaphragmatic movement to almost apneic conditions. HFJV provides extremely low, high-frequency tidal volumes while maintaining effective gas exchange, resulting in minimal chest wall and cardiac movement. Decreasing respiratory motion during an AF ablation procedure allows the operator to more easily improve catheter stability and tissue contact, resulting in better outcomes.^13–15^ In this review article, we discuss important concepts from several studies and review the current literature on HFJV in AF ablation.

## Atrial fibrillation ablation and limitations with traditional techniques

Pulmonary vein (PV) reconnection is considered the most common mechanism of arrhythmia recurrence after PVI. This recurrence occurs due to incomplete tissue destruction during the initial ablation procedure, resulting in the recovery of tissue conduction.^16–18^ The reversible injury is thought, in part, to be the result of poor contact of the catheter with the target tissue for sufficient time during ablation. This challenge can result in the development of inflammation and edema that can hinder further tissue destruction during the procedure. In order to reduce reconnection and the potential for PV “gaps” responsible for arrhythmia recurrence, achievement of permanent PVI through improved or more effective contact and catheter stability should be a primary goal. Over the past decade, different strategies have been developed to improve permanent PVI via creation of transmural and contiguous lesions encircling the circumference of the vein. Much focus has been on improving the ablation catheter technology, including increasing total power, improving power delivery efficiency, contact force (CF) feedback, and balloon technology.^19^ Additionally, adoption of techniques that enhance catheter stability during ablation may improve ablation outcomes.

Studies show that the CF between the catheter and the tissue is affected by respiration and patient movement. More CF and contact time are needed to adequately ablate a moving target. Kumar et al. studied the effect of respiration on CF during ablation of atrial arrhythmia by comparing the CF of ablation lesions delivered under apnea and ventilation.^20^ They found that catheter–tissue CF is critically influenced by respiration during an ablation procedure, with greater CF being observed during apnea.^20^

It is now well known that general anesthesia provides better catheter–tissue contact and thus better procedural success over conscious sedation, owing to improved catheter contact with controlled respiration. In a multicenter trial, Di Biase et al. randomized 129 patients undergoing their first AF ablation procedure to general anesthesia and 128 patients to conscious sedation. At 17 ± 8 months of follow-up, 88 patients assigned to conscious sedation remained free of atrial arrhythmias, as compared to 114 patients randomized to general anesthesia. In this study, all patients with recurrence underwent a second procedure.^21^ Interestingly, during the repeat procedure, 42% of PVs in the conscious sedation group had PV reconnection compared with only 19% in the general anesthesia group, suggesting that general anesthesia results in sustained freedom from recurrent AF due to less PV reconnection.^21^ Although general anesthesia improves AF ablation outcomes and patient tolerance to the procedure by minimizing patient movement, tidal volume changes that occur with intermittent positive pressure ventilation (IPPV) during general anesthesia can create excessive left atrial (LA) movements, particularly in the posterior part, which can potentially impair catheter–tissue CF and thus outcomes of ablation.

## High-frequency jet ventilation

HFJV works by delivering small tidal volumes of oxygen, smaller than the volume of anatomic dead space (1–3 mL/kg), from a high-pressure jet of approximately 35 pounds per square inch (psi) at supraphysiological respiratory rates (> 60 breaths per minute) followed by passive expiration.^22^ HFJV has its own risks and complications, including auto-positive end-expiratory pressure, dynamic hyperinflation secondary to a high respiratory rate and a short expiratory time, pulmonary barotrauma, hemodynamic instability, necrotizing tracheobronchitis, endotracheal tube mucus inspissation, and a variability of cardiac output. Additionally, end-tidal CO_2_ cannot be monitored during HFJV, and intermittent blood gas samples are needed to evaluate blood pH to maintain normocapnia.^23,24^

HFJV was initially used in surgical procedures of the larynx and trachea to keep them fully accessible for surgery. This procedure was achieved by direct placement of a small (14–16 gauge) cannula into the endotracheal tube.^25^ Later, its use expanded to procedures such as extracorporeal shockwave lithotripsy for kidney stones, liver tumor ablations, and AF ablations in subsequent years. These procedures incorporated the technology to minimize motion of the heart, liver, and kidneys. These successes have led to further expansion into new areas. Recently, Gangwani and Sonn presented a case report that utilized HFJV in a successful MitraClip procedure reflecting the potential benefits of this mode of ventilation beyond cardiac ablation procedures.^26^

While it has been demonstrated that general anesthesia with standard ventilation is superior to conscious sedation, both catheter stability and catheter–tissue CF can be further increased by reducing respiratory thoracic excursion via utilizing HFJV.^21^ HFJV leads to nearly static conditions of the chest, which further improves catheter contact, ablation times, and outcomes. Unfortunately, there are no randomized controlled trials comparing general anesthesia with traditional mechanical ventilation versus HFJV for AF ablation procedures. However, a retrospective study from 2006 that compared AF ablation outcomes using HFJV versus general anesthesia with IPPV showed that utilization of HFJV resulted in a shorter procedure duration and a reduced number of ablations required to treat AF.^27^ This endpoint was primarily driven by less LA posterior wall motion, which improved catheter ablation by reducing the electrode dislodgment rate.

HFJV also demonstrates more stable temperature control. It has been shown that IPPV is associated with more ablation electrode temperature variations compared with HFJV due to the variation in the electrode–endocardial contact. In the same study, it was found that HFJV produced less variation in LA volume, pressure, PV blood flow velocity, and posterior LA position than IPPV, which may also explain the better outcomes of AF ablation seen when HFJV is used for creating a favorable biophysical environment for ablation.^27^ Garcia et al. reported that AF ablation using HFJV is of benefit based on time to obtain PVI, fluoroscopy duration, and acute PV reconnection rate.^28^ Hutchinson et al. reported that the contemporary implementation of general anesthesia and HFJV along with the utilization of steerable sheaths and anatomic image integration with computed tomography/magnetic resonance imaging scans resulted in a significantly better one-year freedom from AF and a strong trend toward fewer ablation lesions and shorter time to PVI compared with controls undergoing ablation under conscious sedation. These beneficial results occurred despite a substantially higher prevalence of non-paroxysmal AF, hypertension, elevated body mass index, and larger LA size in the HFJV group.^29^ These findings were linked to a significant reduction in the frequency of both acute and chronic PV reconnection and were attributed to a better and more stable tissue–catheter contact due to controlled breathing patterns and elimination of patient movements. In a recent study by Aizer et al.,^30^ simultaneous modulation of heart rate and respiratory rate by rapid pacing and HFJV, respectively, was shown to significantly improve catheter stability as measured by catheter CF. The authors demonstrated that lesions with rapid pacing and HFJV had better CF and, at the same time, resulted in a decreased proportion of lesions with excessive maximum CF.^30^

Sivasambu et al., in a single-center study, evaluated 84 patients who underwent AF ablation using HFJV compared to 84 matched control patients and followed them up for one year to assess one-year outcomes. Their primary finding was that, at 12 months, freedom from recurrent atrial arrhythmias was significantly higher in the HFJV group compared with the standard ventilation control group (31% vs. 50%; p = 0.012). The authors attributed this finding to better catheter stability, which improved lesion delivery and ablation line integrity. This result was demonstrated by the maintenance of high CF and mean force variability index in the HFJV group compared with the standard ventilation control group. They further reported that even though the use of vasopressors was significantly higher in the HFJV group, complication rates were similar to those in the control groups. Additionally, they demonstrated that utilizing HFJV does not increase complications nor the duration of the procedure.^15^ Elkassabany et al. presented a retrospective study on 188 patients ventilated with HFJV for PVI to evaluate the safety of this mode of ventilation.^31^ In this study, 13 (7%) had to be converted from HFJV to traditional sedation/ventilation techniques due to hypercapnia or hypoxemia. However, the remaining patients completed the ablation procedure under HFJV without any reported complications. Hence, it was concluded that HFJV is considered a safe procedure when used in catheter ablation under general anesthesia.^31^

## Discussion

HFJV, as evidenced by studies summarized in this article, is an effective approach to maintain catheter stability by minimizing patient movement and delivering reliable energy and effective CF to lesions. The studies showed favorable outcomes in terms of long-term AF freedom when HFJV was employed.

If HFJV is considered as a method of ventilation, there are several factors that need to be considered prior to its use. The main issue is the availability of anesthesiologists and staff for general anesthesia. General anesthesia is not widely used due to cost issues and, in certain institutions, due to high patient turnover. Utilizing general anesthesia is time-consuming and adds to the procedure time. Moreover, using HFJV requires a unique ventilator and disposable equipment with the associated additional costs and specialized training for the anesthesia team. While, anecdotally, there are other uses for the ventilator, such as Watchman left atrial appendage closure device implantation and other structural heart cases, its use is still expensive. Furthermore, patient monitoring requires frequent blood draws and blood gas analysis to evaluate oxygenation and CO_2_ levels adding to the complexity of this method. However, this method is increasingly becoming available in different institutions and may become standard of care in the future.

## Conclusion

HFJV during PVI using RF CF catheters is associated with improvement in procedural time and catheter stability. Due to the benefits of HFJV discussed in this article, HFJV support during AF ablation procedures might become standard practice in many institutions in the near future.
